# Biomimetic Approaches in Cardiac Tissue Engineering: Replicating the Native Heart Microenvironment

**DOI:** 10.7759/cureus.43431

**Published:** 2023-08-13

**Authors:** Anoosha Khan, Priya Kumari, Naina Kumari, Usman Shaikh, Chukwuyem Ekhator, Raghu Halappa Nagaraj, Vikas Yadav, Aimen Waqar Khan, Slobodan Lazarevic, Bishal Bharati, Gautham Lakshmipriya Vetrivendan, Asmita Mulmi, Hana Mohamed, Ashraf Ullah, Bijan Kadel, Sophia B Bellegarde, Abdur Rehman

**Affiliations:** 1 Medicine, Dow University of Health Sciences, Karachi, PAK; 2 Medicine, Jinnah Postgraduate Medical Centre, Karachi, PAK; 3 Dow Medical College, Dow University of Health Sciences, Karachi, PAK; 4 Neuro-Oncology, New York Institute of Technology, College of Osteopathic Medicine, Old Westbury, USA; 5 Surgery, Avalon University School of Medicine, Willemstad, CUW; 6 Internal Medicine, Pandit Bhagwat Dayal Sharma Post Graduate Institute of Medical Sciences, Rohtak, IND; 7 Medicine, Jinnah Sindh Medical University, Karachi, PAK; 8 Internal Medicine, University of Niš Faculty of Medicine, Niš, SRB; 9 Internal Medicine, Nepal Medical College, Kathmandu, NPL; 10 Medicine, Karpaga Vinayaga Institute of Medical Sciences & Research Center, Kancheepuram, IND; 11 Medicine, TMSS Medical College, Bogura, BGD; 12 Medicine, United Nations Study & Understanding, The International Academy, Khartoum, SDN; 13 Medicine, Elrazi University, Khartoum, SDN; 14 Internal Medicine, Mayo Hospital, Lahore, PAK; 15 Internal Medicine, Nepal Medical College and Teaching Hospital, Kathmandu, NPL; 16 Pathology and Laboratory Medicine, American University of Antigua, St. John's, ATG; 17 Surgery, Mayo Hospital, Lahore, PAK

**Keywords:** biomimetic microenvironment, review, cardiac regeneration, microenvironment, heart, cardiac

## Abstract

Cardiovascular diseases, including heart failure, pose significant challenges in medical practice, necessitating innovative approaches for cardiac repair and regeneration. Cardiac tissue engineering has emerged as a promising solution, aiming to develop functional and physiologically relevant cardiac tissue constructs. Replicating the native heart microenvironment, with its complex and dynamic milieu necessary for cardiac tissue growth and function, is crucial in tissue engineering. Biomimetic strategies that closely mimic the natural heart microenvironment have gained significant interest due to their potential to enhance synthetic cardiac tissue functionality and therapeutic applicability. Biomimetic approaches focus on mimicking biochemical cues, mechanical stimuli, coordinated electrical signaling, and cell-cell/cell-matrix interactions of cardiac tissue. By combining bioactive ligands, controlled delivery systems, appropriate biomaterial characteristics, electrical signals, and strategies to enhance cell interactions, biomimetic approaches provide a more physiologically relevant environment for tissue growth. The replication of the native cardiac microenvironment enables precise regulation of cellular responses, tissue remodeling, and the development of functional cardiac tissue constructs. Challenges and future directions include refining complex biochemical signaling networks, paracrine signaling, synchronized electrical networks, and cell-cell/cell-matrix interactions. Advancements in biomimetic approaches hold great promise for cardiovascular regenerative medicine, offering potential therapeutic strategies and revolutionizing cardiac disease modeling. These approaches contribute to the development of more effective treatments, personalized medicine, and improved patient outcomes. Ongoing research and innovation in biomimetic approaches have the potential to revolutionize regenerative medicine and cardiac disease modeling by replicating the native heart microenvironment, advancing functional cardiac tissue engineering, and improving patient outcomes.

## Introduction and background

Cardiovascular diseases, including heart failure, remain a major worldwide medical challenge, necessitating innovative strategies for cardiac repair and regeneration. Over the past decade, cardiac tissue engineering has emerged as a promising approach to address the limitations of conventional therapies by aiming to develop functional and physiologically relevant cardiac tissue constructs [1,2]. Replicating the native heart microenvironment, which offers a complex and dynamic milieu required for the growth, function, and homeostasis of cardiac tissue, is a crucial component of effective tissue engineering. Due to their potential to improve the functioning and therapeutic applicability of synthetic cardiac tissues, biomimetic strategies that closely resemble the natural heart microenvironment have drawn a lot of interest [3].

The native cardiac tissue comprises different cell types and an intricate extracellular matrix, forming a microenvironment that regulates cellular behavior, tissue remodeling, and electrical conduction. Biomimetic approaches aim to replicate this microenvironment to develop functional cardiac tissue constructs. These strategies seek to mimic the natural biochemical cues, mechanical stimulation, coordinated electrical signaling, and cell-cell/cell-matrix interactions of cardiac tissue [4,5]. Biomimetic approaches offer a more physiologically realistic environment for tissue growth by combining bioactive ligands, regulated delivery systems, suitable biomaterial characteristics, electrical signals, and strategies for increasing cell interactions [3]. These methods allow for precise regulation of cellular responses, tissue remodeling, and the development of constructions of functioning heart tissue.

Additionally, imitating mechanical signals through suitable biomaterial characteristics and scaffold architecture affects cellular activities, including adhesion, migration, proliferation, and tissue remodeling, improving the performance of synthetic cardiac structures [6,7]. The development and performance of synthetic tissues are further enhanced by including electrical signals and encouraging synchronized electrical networks. Gap junctions, adhesion molecules, and the extracellular matrix, which promote cell-cell and cell-matrix interactions, are also essential for biomimetic methods. The formation of functional cardiac tissue constructions can be aided by co-culture strategies, spatial patterning technologies, and biomaterials. Overall, the replication of the native cardiac microenvironment using biomimetic methods holds great promise for improving therapeutic approaches for cardiovascular diseases and revolutionizing cardiac regenerative medicine through continual research and innovation.

## Review

Native cardiac microenvironment

The native cardiac microenvironment provides a complex and dynamic milieu that is essential for the development, function, and homeostasis of cardiac tissue. In biomimetic techniques for cardiac tissue development, it is essential to replicate the natural heart microenvironment. The main elements of the native cardiac microenvironment will be discussed in this part, along with the biochemical, mechanical, and electrical cues that are present and affect cellular activity and tissue function.

Numerous cell types, including cardiomyocytes, endothelial cells, fibroblasts, and immune cells, make up the native heart tissue [8]. Within the heart, these cells are arranged in a highly organized manner to produce the extracellular matrix (ECM), blood vessels, and myocardial fibers. The ECM supports structural integrity by anchoring cells and promoting interactions between cells and the matrix. It is made up of proteoglycans and glycosaminoglycans, as well as proteins, including collagen, elastin, and laminin. The native heart tissue's functional characteristics are influenced by the organization and distribution of its cells and ECM components [8].

Growth factors, cytokines, and other signaling molecules that control cell behavior and tissue formation are among the biochemical cues present in the native cardiac microenvironment. Growth factors play important roles in heart development, angiogenesis, and tissue healing. Examples include transforming growth factor-beta (TGF-β), insulin-like growth factor (IGF), and vascular endothelial growth factor (VEGF). The biochemical cues that affect cellular responses and tissue function are also influenced by paracrine signaling between various cell types in the heart tissue [9].

Cell behavior and tissue function are significantly influenced by the mechanical characteristics of the heart, which include stiffness, elasticity, and contractility. The contraction-relaxation cycle of the heart causes cardiomyocytes to undergo cyclic mechanical loading. By giving resistance to deformation and conveying mechanical forces, the ECM serves as a mechanical cue. Cell adhesion, migration, proliferation, and differentiation are all influenced by mechanical signals. Additionally controlling gene expression and tissue remodeling processes are the dynamic mechanical stresses [10,11].

The correct operation of the heart depends on synchronized electrical signaling. Cardiomyocytes are largely responsible for producing and transmitting electrical cues in the native cardiac milieu through the creation of action potentials and intercellular electrical coupling. Electrical current can travel via gap junctions between neighboring cardiomyocytes, synchronizing cell activity and enabling coordinated contraction. Electrical stimuli affect the excitability of cardiac tissue cells, the spread of action potentials, and the development of functional syncytia [12].

The native cardiac microenvironment is fundamentally composed of cell-cell and cell-matrix interactions. Gap junctions serve as a bridge for direct cell-to-cell connections between cardiomyocytes, enabling synchronized electrical and mechanical activity. Other cell types that interact with cardiomyocytes and support tissue function include endothelial cells and fibroblasts. Cells and the ECM adhere to one another through integrin-mediated processes in cell-matrix interactions. Cell migration, proliferation, and differentiation are all governed by these interactions [13,14].

Replicating the native heart microenvironment in biomimetic approaches involves understanding and incorporating these key components and cues. Biomaterials can be created with bioactive ligands and cell attachment sites to imitate the structure and makeup of the ECM. The reproduction of biochemical signals, like growth factors, to control cellular behavior is possible, thanks to controlled delivery methods. By simulating the mechanical characteristics of the natural cardiac microenvironment, scaffold architecture can give cells mechanical signals. The electrical cues seen in the natural cardiac microenvironment can be replicated using electrical stimulation methods and electroconductive materials. Co-culture methods and spatial patterning approaches, in addition, aid in the biomimetic reproduction of the natural cardiac milieu by increasing cell-cell and cell-matrix interactions. Biomimetic approaches in cardiac tissue engineering can offer a more physiologically relevant environment for the creation of functional cardiac tissue constructs by comprehending and incorporating the biochemical, mechanical, and electrical cues as well as cell-cell and cell-matrix interactions present in the native heart microenvironment.

Biomimetic strategies for biochemical replication

The native cardiac microenvironment is heavily influenced by biochemical signals, which control cellular activity and tissue growth. To successfully design heart tissue, these signals must be replicated. This section discusses several biomimetic techniques used to replicate the biochemical features of the natural cardiac microenvironment.

Cells get structural support and molecular signaling signals from the ECM. Biomaterials can be created to imitate the structure, composition, and mechanical characteristics of natural ECM [15]. Scaffolds that imitate the ECM have been made using both synthetic and natural polymers, including collagen, fibrin, and hyaluronic acid. These biomaterials have the ability to offer bioactive ligands, control the release of signaling molecules, and provide cell adhesion sites, which promote cellular attachment, migration, and differentiation [7,16].

Growth factors are essential for angiogenesis, tissue healing, and heart development. Controlled growth factor administration is used in biomimetic methods to mimic the signaling pathways seen in the natural cardiac microenvironment [17]. Hydrogels, microparticles, and nanoparticles are examples of controlled-release systems that may be utilized to provide growth factors locally and over time. In designed cardiac tissue constructions, this enables precise spatiotemporal regulation of growth factor release, boosting cell proliferation, migration, and differentiation [18-20].

Cell-cell interactions are fundamental for the formation of functional cardiac tissue. To encourage cell-cell interactions, cardiac cells are seeded into artificial constructions utilizing biomimetic methodologies [21]. Co-culture systems, which combine many cell types (including cardiomyocytes, endothelial cells, and fibroblasts), simulate the biological makeup of heart tissue in vivo [22]. Additionally, the three-dimensional (3D) organization and interactions seen in the natural cardiac milieu may be replicated using spatial patterning techniques and microfluidic platforms [23].

Bioactive compounds that mimic the natural biochemical signals of the cardiac microenvironment can be created using molecular and genetic engineering approaches. This may entail genetically altering cells to overexpress certain cytokines, ECM proteins, or growth factors. Additionally, gene editing techniques, such as CRISPR/Cas9, can be utilized to introduce precise genetic modifications in cells, enhancing their regenerative potential. By genetically engineering cells or introducing exogenous gene products, the expression of key biomolecules can be controlled, promoting cell survival, tissue maturation, and functional integration within the engineered cardiac tissue [24,25].

These biomimetic strategies for biochemical replication are critical in creating engineered cardiac tissues that closely mimic the native heart microenvironment. Researchers can re-create the complex biochemical cues required for controlling cell behavior, promoting tissue development, and achieving functional cardiac tissue engineering by using biomaterials, growth factor delivery systems, cell seeding strategies, and molecular/genetic engineering approaches. Overall, these biomimetic techniques not only enhance cardiac tissue engineering but also show promise for regenerative therapeutics, disease modeling, and drug screening platforms that have the potential to revolutionize the field of cardiology.

Biomimetic strategies for mechanical replication

The mechanical properties of the native heart microenvironment play a crucial role in maintaining tissue integrity and regulating cellular behavior. It is crucial to reproduce these mechanical stimuli for successful cardiac tissue engineering [3]. This section examines biomimetic techniques for replicating the mechanical aspects of the natural cardiac microenvironment.

The design and construction of the scaffold are crucial factors in simulating the mechanical characteristics of heart tissue. Biomimetic scaffolds are designed to resemble the natural stiffness, elasticity, and microstructure of the heart. Scaffolds with adjustable mechanical characteristics using a range of materials and manufacturing methods have been developed for this purpose. For instance, the elasticity of heart tissue may be mimicked using hydrogels made of alginate and collagen-based polymers [18,26]. Additionally, scaffolds with regulated pore sizes, fiber orientations, and mechanical characteristics have been made using methods like electrospinning and 3D printing, closely resembling the natural cardiac ECM [4,27,28].

Biomechanical stimulation is critical for the maturation of engineered cardiac tissue. To promote cellular alignment, contractility, and tissue organization, a variety of approaches have been used to deliver mechanical pressures that resemble those encountered by natural heart tissue. Physiological mechanical stimuli have been applied to the synthetic tissue utilizing stretching, cyclic mechanical strain, and mechanical conditioning in bioreactors [10]. These methods have been found to increase tissue contractile characteristics, cell alignment along the main axis of stress, and the production of cardiac-specific proteins. The ECM is also influenced by biomechanical stimulation, which furthers the mechanical maturation of the synthetic heart tissue [29-31].

The contractile function of cardiac tissue depends on the anisotropic nature of the tissue, which is characterized by the preferred alignment of cells and ECM. Engineering anisotropic cardiac tissue using biomimetic techniques entails developing aligned cellular and matrix structures [32]. Microcontact printing, micropatterning, and microfluidics are examples of techniques that have been used to direct cell alignment and regulate the ECM's organization. These methods enable the reproduction of the anisotropic mechanical characteristics seen in the native heart by offering signals for directed cell growth and matrix deposition. It is quite possible to create functioning cardiac constructions and patches using anisotropic cardiac tissue engineering that mimic the mechanical properties of the heart muscle [33-35].

By employing scaffold design and fabrication techniques, biomechanical stimulation methods, and engineering anisotropic tissue, researchers aim to replicate the mechanical properties of the native heart microenvironment. These biomimetic strategies enable the development of engineered cardiac tissue that closely resembles the functional and structural characteristics of native cardiac tissue. The incorporation of these mechanical cues not only contributes to the maturation and functionality of engineered tissue but also holds promise for applications such as cardiac repair, disease modeling, and drug screening.

Biomimetic strategies for electrical replication

The electrical properties of the native heart microenvironment are crucial for coordinating the rhythmic contraction of cardiac tissue. It is crucial to replicate these electrical signals for the effective development of functioning heart tissue. This section examines biomimetic techniques used to replicate the electrical characteristics of the natural cardiac microenvironment.

Biomaterials with built-in electroconductive characteristics are used to mimic the electrical conductivity of native heart tissue. Scaffolds are infused with electroconductive elements, including carbon nanotubes, graphene, and conductive polymers, to create a conductive network across the constructed tissue [36]. These materials imitate the electrical conductivity seen in the natural heart and help electrical messages spread. Electroconductive scaffolds are used to facilitate synchronized electrical activity inside the designed tissue and to encourage cell-cell communication [37,38].

Electrical stimulation techniques are employed to promote cell alignment and enhance contractile function in engineered cardiac tissue. This stimulation encourages cardiomyocyte alignment, which results in better tissue organization and increased contractile characteristics [39,40]. Additionally, electrical stimulation affects cellular behavior, including cell division, proliferation, and gene expression, promoting tissue maturation and functionality.

To enhance electrical coupling within engineered cardiac tissue, electroactive cells, and nanomaterials are integrated into the constructs. Cardiomyocytes or cardiac progenitor cells produced from stem cells are examples of electroactive cells, which have electrical characteristics of their own and can contribute to the electrical functionality of the tissue [41]. Additionally, metallic nanoparticles and nanowires can be added to nanomaterials to improve electrical connections between cells and scaffolding materials. Within the synthetic tissue, these nanoparticles boost cell-scaffold interactions, encourage cell adhesion, and improve electrical signal transmission [42].

The electrical properties of the natural cardiac microenvironment can be replicated by the use of electroconductive materials, electrical stimulation techniques, electroactive cells, and nanomaterials. These biomimetic techniques not only support electrical functioning but also aid in the formation of cardiac-like electrical behavior inside the engineered tissue, tissue maturation, and synchronization of contractile activity.

Evaluation of biomimetic cardiac tissue constructs

The effective development of biomimetic cardiac tissue constructs implies rigorous and thorough examination to make sure that the engineered tissues closely approximate the native heart microenvironment [43]. The main criteria for assessing the development of biomimetic heart tissue are covered in this section.

The evaluation of biomimetic cardiac tissue constructs involves assessing the presence and effectiveness of biochemical cues and signaling pathways that mimic those in the native heart microenvironment. To examine the expression of certain proteins, growth factors, and signaling molecules, techniques including immunohistochemistry, gene expression analysis, and proteomics can be used. The existence of pertinent biochemical cues and their appropriate localization within the altered tissues can be confirmed by these investigations. The signaling activity and downstream effects of the biomolecules included in the construct may also be assessed using functional and bioactivity assays [44,45].

The functioning of cardiac tissue is dependent on its mechanical characteristics, which include stiffness, elasticity, and contractile strength [46]. Tensile testing, atomic force microscopy, and rheological analysis are some of the techniques that may be used to assess the mechanical characteristics of biomimetic heart tissue structures. These techniques offer quantitative information on the mechanical behavior of the tissue, such as its reaction to forces, deformation, and viscoelastic characteristics [47,48]. The resemblance and usefulness of the created structures may be evaluated by comparing their mechanical characteristics to those of natural heart tissue.

One of the key features of cardiac tissue is its ability to propagate electrical signals and exhibit synchronized contractile behavior. Assessing the electrical coupling and contractile characteristics of biomimetic heart tissue constructions is necessary for functional assessment. Electrical conduction and action potentials inside the tissue constructs can be determined by electrode arrays, microelectrode arrays, or optical mapping methods [49,50]. Functional evaluation can also include analyzing contractile behavior using techniques such as video microscopy, force measurements, or calcium imaging [51]. These assessments provide insights into the tissue's ability to propagate electrical signals and exhibit coordinated contractions, resembling the functionality of native cardiac tissue.

To further evaluate the performance and integration of biomimetic cardiac tissue constructs, both in vitro and in vivo models are utilized. In vitro models provide controlled evaluations of cell behavior, tissue functioning, and stimulus responses. These models can make use of tissue-on-a-chip platforms, microfluidic devices, or bioreactors, which offer a controlled setting for examining tissue function [52,53]. Animal models and other in vivo simulations enable a more thorough assessment of tissue integration, vascularization, and physiological response. The long-term survivability, functioning, and possible immunological reactions of the synthetic cardiac tissue constructs can be better understood by in vivo studies.

Advancements and challenges

Biomimetic approaches in cardiac tissue engineering have witnessed significant advancements in recent years, bringing us closer to achieving functional and physiologically relevant cardiac tissue constructs. However, despite these developments, there are still a number of issues that must be resolved before biomimetic techniques may be successfully applied in clinical settings [45].

Advancements in technology and the development of innovative biomaterials have greatly enhanced the biomimetic potential of cardiac tissue engineering. For instance, the exact spatial organization of cells, biomaterials, and growth factors is made possible by 3D bioprinting techniques, which makes it easier to create intricate heart tissue architectures [54]. Additionally, the range of biomaterial possibilities for simulating the natural cardiac microenvironment has increased with the development of bioactive and smart materials with controllable features. These developments offer novel opportunities to improve biomimicry and functionality in synthetic cardiac tissues [55].

Microfluidic systems and organ-on-a-chip platforms offer unique advantages in replicating the physiological conditions and interactions found in the native heart microenvironment. To precisely imitate the complex circumstances of cardiac tissue, these technologies enable the integration of many cell types, regulated fluid flow, and dynamic mechanical stimulation. Microfluidics may be used to build physiologically realistic cardiac models that faithfully depict the intricate interactions between cells, the vasculature, and extracellular stimuli. These systems offer a potent tool for applications such as drug screening and personalized medicine [56,57].

Immunomodulation plays a crucial role in the successful integration of engineered cardiac tissues. The immune response has a considerable effect on the long-term viability, functioning, and survival of tissues. Numerous methods of modulating the immune system are being investigated, including the use of immunosuppressive drugs, immunomodulatory nanomaterials, and immune cell engineering. These approaches seek to dampen the immune response, minimize inflammation, and stimulate tissue integration. Advancements in immunomodulatory strategies are essential for enhancing the compatibility and integration of biomimetic cardiac constructs in the host tissue [58,59].

One of the key challenges in biomimetic cardiac tissue engineering is the scalability of the fabrication techniques and the translation of these approaches to clinical applications. For broad application, biomimetic cardiac tissue components must be developed that can be mass-produced and adhere to regulatory requirements [60]. To meet this challenge, approaches including high-throughput production techniques, scalable bioreactor systems, and standard protocols are being investigated [61]. Additionally, to assure safety, effectiveness, and regulatory compliance, the translation of biomimetic cardiac tissue engineering into clinical applications necessitates close cooperation between researchers, clinicians, and regulatory authorities.

While significant advancements have been made in the field of biomimetic cardiac tissue engineering, several challenges remain. The issues that need to be addressed include standardizing production methods, optimizing functional and structural maturation, and ensuring the long-term stability of synthetic cardiac tissues [60]. Additionally, addressing the complexities of vascularization, achieving electromechanical integration, and ensuring long-term functionality and durability pose significant hurdles.

Future directions and outlook

The field of cardiac tissue engineering has made significant progress in replicating the native heart microenvironment and engineering functional cardiac tissue. However, there are several fascinating future directions and potential advancements that hold great promise for further enhancing biomimicry and advancing the field. The prospective effects of four important future directions on cardiac tissue engineering are covered in this section.

Future research should focus on integrating multiscale and multiparametric approaches to further replicate the complexity of the native heart microenvironment. This entails taking into account many organizational levels, including molecular, cellular, tissue, and organ scales, as well as incorporating different characteristics like biochemical, mechanical, electrical, and spatial inputs. Combining these methods enables researchers to develop more complex and physiologically accurate cardiac tissue models that more accurately mimic the natural cardiac environment.

Personalized medicine aims to tailor medical treatments to an individual's unique characteristics, and integrating patient-derived cells in cardiac tissue engineering holds great potential in this regard [62]. The development of patient-specific cardiac cells, which are more adequately suited to represent the individual's disease phenotype and responsiveness to treatment, is made possible by the use of induced pluripotent stem cells (iPSCs) produced from patients. For the improvement of patient outcomes in cardiac tissue engineering, this personalized approach can assist in the creation of targeted medicines, drug screening platforms, and prediction models.

Engineering functioning heart tissue requires precise spatial organization. The exact deposition of cells, biomaterials, and bioactive compounds in intricate 3D constructions is now possible because of advancements in biofabrication techniques like 3D bioprinting. The ability to recreate the complicated structures and organization present in the natural heart is made possible by the integration of bioprinting with cutting-edge imaging and computational modeling [63]. This skill makes it possible to develop biomimetic heart tissue constructions with predetermined microenvironments and functional physiological properties.

Vascularization is essential for the survival and functionality of engineered cardiac tissues. A critical future aim is the creation of methods for bioengineering vascular networks within the structures. To stimulate the growth of blood vessels, this may entail the inclusion of endothelial cells and the use of biomaterials with angiogenic capabilities. Additionally, complex perfusable vascular networks that can sustain nutrition and oxygen transport to the engineered heart tissue may be created using methods such as sacrificial biofabrication and microfluidics [64]. Larger and more metabolically active tissue constructions will be made possible by the successful integration of functional vascular networks, increasing their physiological significance and therapeutic applicability.

## Conclusions

Biomimetic approaches have made remarkable progress in imitating the natural heart microenvironment and propelling the field of cardiac tissue engineering forward. Notable breakthroughs in this area involve the integration of bioactive substances and controlled delivery techniques to replicate the biochemical signals found in the native heart. These approaches aim to precisely regulate cellular responses and tissue growth, ultimately striving to develop cardiac tissue constructs that closely resemble the physiological characteristics of the heart. The implications of these advancements in biomimetic approaches are vast. They hold immense promise for regenerative medicine, offering potential therapeutic strategies for cardiovascular diseases. By recreating the complexity and dynamics of the native cardiac microenvironment, these approaches can contribute to the development of more effective treatments and interventions. Moreover, biomimetic cardiac tissue constructs can serve as valuable tools for cardiac disease modeling, enabling researchers to study disease mechanisms, evaluate drug efficacy, and explore personalized medicine approaches. The ongoing research and innovation in biomimetic approaches have the potential to revolutionize regenerative medicine and cardiac disease modeling. By replicating the native heart microenvironment, advancing functional cardiac tissue engineering, and improving patient outcomes, these approaches pave the way for significant advancements in the field.
